# A Graphene-Based Enzymatic Biosensor Using a Common-Gate Field-Effect Transistor for L-Lactic Acid Detection in Blood Plasma Samples

**DOI:** 10.3390/s21051852

**Published:** 2021-03-06

**Authors:** Ariadna Schuck, Hyo Eun Kim, Júlia Konzen Moreira, Priscila Schmidt Lora, Yong-Sang Kim

**Affiliations:** 1 Department of Electrical and Computer Engineering, Sungkyunkwan University, Suwon 16419, Korea; arischuck@g.skku.edu (A.S.); hyoeun0707@naver.com (H.E.K.); 2 Graduate Program in Nursing, Universidade do Vale do Rio dos Sinos, 93022-750 São Leopoldo, Brazil; juliakonzenmoreira@hotmail.com (J.K.M.); plora@unisinos.br (P.S.L.)

**Keywords:** lactate, lactate dehydrogenase, graphene, field-effect transistor, biosensor

## Abstract

Lactate is an important organic molecule that is produced in excess during anaerobic metabolism when oxygen is absent in the human organism. The concentration of this substance in the body can be related to several medical conditions, such as hemorrhage, respiratory failure, and ischemia. Herein, we describe a graphene-based lactate biosensor to detect the concentrations of L-lactic acid in different fluids (buffer solution and plasma). The active surface (graphene) of the device was functionalized with lactate dehydrogenase enzyme using different substances (Nafion, chitosan, and glutaraldehyde) to guarantee stability and increase selectivity. The devices presented linear responses for the concentration ranges tested in the different fluids. An interference study was performed using ascorbic acid, uric acid, and glucose, and there was a minimum variation in the Dirac point voltage during detection of lactate in any of the samples. The stability of the devices was verified at up to 50 days while kept in a dry box at room temperature, and device operation was stable until 12 days. This study demonstrated graphene performance to monitor L-lactic acid production in human samples, indicating that this material can be implemented in more simple and low-cost devices, such as flexible sensors, for point-of-care applications.

## 1. Introduction

Lactate is present in various biological processes and medical conditions, and it has an important role in the anaerobic metabolic pathway [1,2]. Usually, the lactate concentration increases when there is a deficit of oxygen [1,3,4]. It also can be related to higher energy demand and it is a common target for monitoring of physical exercise routine [5]. However, the elevated concentration of lactate is also related to tissue hypoxia and accelerated aerobic metabolism, being a prognosis marker for different disorders, e.g., septic shock and left ventricular failure [5,6,7]. It is essential to detect the lactic acidosis level in the patient to accelerate the treatment outcome and decrease the mortality rate caused by sepsis [5]. There are many methods to detect lactate, but they are still inaccurate with a long turnaround time and sample limitation. Although, sepsis is still the major cause of mortality in the world and a proper point-of-care device is necessary to obtain a faster and precise diagnosis to prevent this immunosuppressive condition [7].

Most of the reported biosensors used to detect lactate concentration depend on a specific enzyme to catalyze the reaction and enhance the selectivity; the product of the reaction is usually proportional to the analyte concentration [1,5,8,9,10,11]. Lactate oxidase (LOx) and lactate dehydrogenase (LDH) are the most common enzymes used as catalysts to bind the reactant molecule and catalyze the enzymatic reaction resulting in the product molecule [3,5,9,12,13,14,15]. The lactate oxidase enzyme is responsible for the production of pyruvate and hydrogen peroxide through the catalyzation of lactate and oxygen [12,16,17]. However, the LOx is unstable since it loses its activity in a short time [8,18]. Contrastingly, the lactate dehydrogenase enzyme catalyzes lactic acid into pyruvic acid [2,3,19]. In anaerobic metabolism, pyruvic acid is converted to lactic acid when there is a deficit of oxygen during the glycolysis process [3]. However, the lactate dehydrogenase enzyme can act over other substrate molecules in certain conditions, increasing the final product [3,19]. Considering this, it is necessary to block the substances in body fluids that can cause interference in the signal response, resulting in false-positive results. Some studies have identified chemicals to immobilize the enzyme in the active site and block the interfering substances, such as ascorbic and uric acids [9,20,21,22,23]. 

In the clinical environment, lactate concentration is commonly measured using a blood gas analyzer that is large bench-top point-of-care (POC) equipment and it has certain limitations regarding processing time and sample preparation [6,8,24]. The quantification of lactate has been widely explored in the literature; there are several devices reported as a lactate sensor using different measurement techniques, device structure, materials, and biological fluids [1,5]. Most of the studies are focusing on sweat and saliva samples to produce non-invasive devices [1,8,14,15,23,25,26], but these biological fluids present a bad signal response due to contamination, pH values, and low concentration of the target [27]. Using blood samples as electrolyte guarantees the detection of lactate even in small concentrations without a high number of interferents, and it is easy to collect in emergencies, especially in small volumes [28,29]. In healthy humans, the lactate concentration in blood is from 0.6 to 2.0 mM, but in clinical conditions, such as sepsis shock, the level of this substance can increase above4 mM [9,30,31].

Graphene is a single-layer material with a large surface area and paramount electronic and chemical properties that can increase the sensitivity and specificity of a biosensor when applied as an active layer [12,32,33,34,35,36,37]. A graphene-based field-effect transistor (GFET) has been applied as sensors by using bioreceptors that were immobilized over its surface while providing a controlled environment for detection and monitoring of physiological/biological processes [33,34,37,38,39]. The electrical response in a GFET is detected due to the presence of the charged biomolecules that were adsorbed on the surface of the graphene when an electric field is applied [32,34,40,41]. The graphene is commonly coupled with another conductive material, e.g., gold or platinum nanoparticles, to increase the sensitivity of the sensor [19,42]. But it also was used as an electrode in flexible bionanosensors to improve the sensing properties [12,16]. In the literature, there are a few studies using graphene and graphene oxide for the detection of lactate concentration [12,19,20,25]. Most of the graphene-based devices used electrochemical methods to detect lactate, but they barely explored the transfer characteristics of this material [4,5,25,42]. Some of the graphene-based devices presented a limitation regarding the detection range [12,20], while others barely presented the performance of the device considering its stabilization, selectivity, sensitivity, or reliability [12,20,25,43]. 

In this study, we present a multiplexed solution-gated graphene-based field-effect transistor sensor to determine the concentration of lactate in different fluids. The lactate was detected using the LDH enzyme to catalyze the chemical reaction in the biological fluid. The enzyme was immobilized using different substances to guarantee stability and selectivity over the active layer. The transfer characteristics of the common-gate GFET were measured for each concentration in different electrolytes (buffer solution and blood plasma). The selectivity was verified using interference substances (uric acid, ascorbic acid, and glucose), and the stability of the GFET devices was monitored for up to 50 days.

## 2. Materials and Methods

### 2.1. Materials

AZ-1512, SU-8 2075, hexamethyldisilazane (HMDS), and (1-methoxy-2-propyl) acetate (SU-8 developer) were purchased from MicroChem Corp. (Westborough, MA, USA). Polydimethylsiloxane (PDMS) (Sylgard 184 A/B) was obtained from Dow Corning (Seoul, Korea). Graphene layers grown by chemical vapor deposition (CVD) were acquired from Graphenea (San Sebastián, Spain). Human plasma, uric acid, ascorbic acid, iron (III) chloride powder, and phosphate-buffered saline were supplied by Sigma-Aldrich Corp. (St. Louis, MO, USA). Illustrations were created with BioRender.com.

### 2.2. Fabrication of the Common-Gate Graphene-Based Transistor

**Figure 1:**
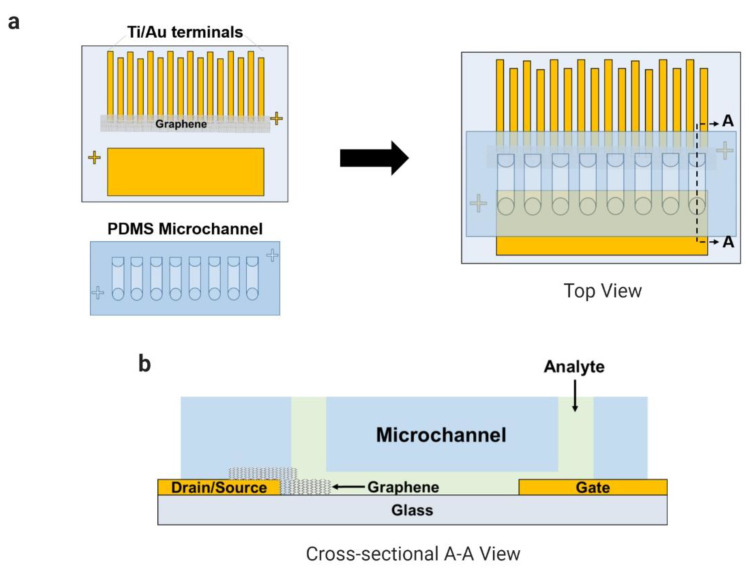
Microchannels integrated with the electrode array (eight pairs) of the common-gate graphene-based field-effect transistor (GFET) sensor to detect L-lactic acid in human samples: (**a**) top view and (**b**) cross-sectional A–A view.

The fabrication of the coplanar electrodes on a glass substrate was reported in our previous works (Han et al., 2017; Kim et al., 2019; Schuck et al., 2020). Briefly, the glass substrate was spin-coated with the adhesion promoter (HMDS) and with the positive photoresist (AZ-1512). The electrodes were patterned using a photomask and ultraviolet radiation over the glass by a mask aligner (MA-6, Karl-Suss). The titanium and the gold layers were deposited over the glass using a vacuum thermal evaporator system (Evaporation System SHE-6T-350D). The CVD-grown graphene coated with a PDMS layer was transferred onto the region between the eight pairs of electrodes (source and drain) after the copper layer was etched away, and the supportive layer (PDMS) was detached from the graphene during an acetone bath. The PDMS microfluidic channels containing eight channels were bonded onto the substrate by UV–ozone treatment. In Figure 1, the final device is presented with a multiplexed common-gate GFET structure integrated with PDMS microchannels.

### 2.3. Enzyme Immobilization and Sample Preparation

**Figure 2:**
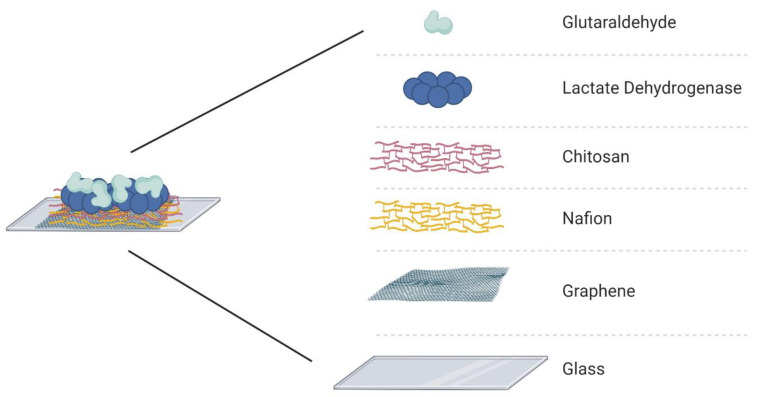
Immobilization matrix to support the enzyme (lactate dehydrogenase) on the surface of the active layer (graphene) of the GFET biosensor.

The electrode array was assembled with PDMS channels and the active layer surface was treated with different substances to guarantee stability and selectivity of the device. The enzymatic matrix was fabricated using Nafion, chitosan, lactate dehydrogenase enzyme, and glutaraldehyde. First, 3 µL of Nafion (NA) was drop-casted over the graphene for 1 h, after which the channels were washed with phosphate-buffered saline (PBS). Nafion is a sulfonate fluoropolymer–copolymer and is used as a membrane to block anionic interference species, such as ascorbic acid [5,8,44,45,46]. Next, chitosan (CHI) was drop-casted (5 µL) over the graphene/Nafion surface and allowed to dry for 1 h, after which the channels were washed with the PBS solution. The presence of chitosan allows immobilization of LDH and guarantees its catalytic performance [5,13,19]. The LDH enzyme (5 µL) was injected into the channels and dried at room temperature for 1 hour. The last component of the immobilization matrix is glutaraldehyde (GA), which was deposited over the surface of the active layer; GA is a crosslinking reagent that can increase the stability of the immobilized enzyme [5]. The final structure of the immobilization matrix is presented in Figure 2. The deposition order of each biochemical was based on the literature to guarantee high binding-stability and block the possible interferents [5,14,23,47]. Nafion was selected as the first layer over graphene to work as the main protective layer, and it can repel the molecules with the same electrostatic charge [13,48,49]. These chemicals were selected considering their effects on the enzyme and the graphene surface. It is important to avoid the doping effect of graphene, except for the product of the chemical reaction that the LDH will catalyze when in contact with the L-lactic acid.

### 2.4. Measurement, Storage, and Analysis

The transfer characteristics of the graphene layer were verified using an HP Agilent 4145B Semiconductor Parameter Analyzer (Hewlett-Packard Company, Tokyo, Japan) while using the buffer solution (PBS) as an analyte. The Dirac point voltage (V_Dirac_) of the bare graphene was determined considering the lowest drain current (I_D_) measured while applying a constant bias (V_D_) on the drain terminal of 0.4 V and varying the bias (V_G_) applied to the gate. The microchannels were cleaned with deionized water and dried in an oven after the characterization of the bare devices. Then, the enzyme was immobilized following the steps mentioned in the previous subsection to produce the immobilization matrix. 

**Figure 3:**
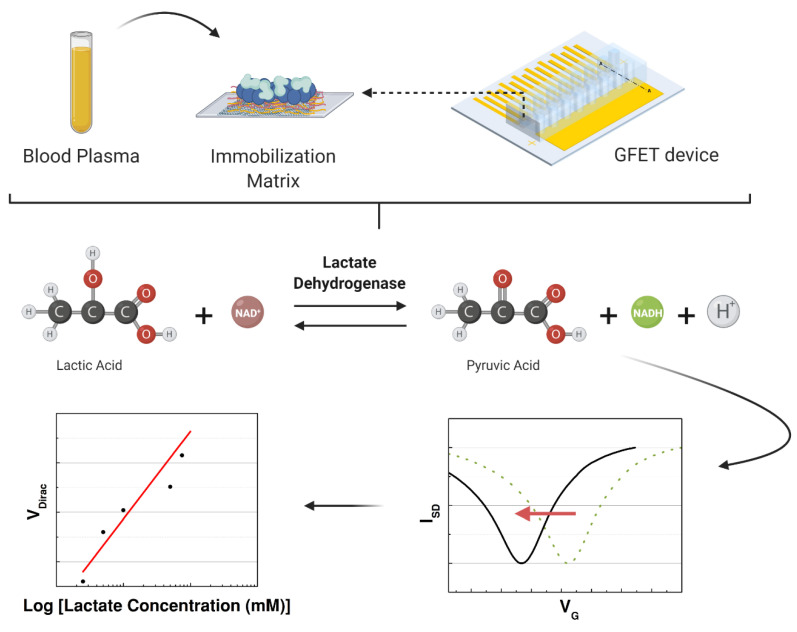
Schematic of the study performed to detect the concentration of lactate in the samples that were injected in the microchannels over the immobilization matrix while measuring the transfer characteristics of the graphene-based device.

After immobilization, all the fabricated devices were maintained at room temperature inside a dry box until the experiment. On day 1, the devices were tested with a PBS solution with different concentrations of L-lactic acid. Immobilization was evaluated with and without the presence of each compound combined with the lactate dehydrogenase. The devices were fabricated to be tested for 50 days in the same conditions using the best immobilization matrix to support the LDH enzyme. When the capability of the sensor to detect L-lactic acid was confirmed, the graphene-based sensor was used to detect the lactate levels in human samples (blood plasma) to focus on clinical applications. A schematic of the experiment is illustrated in Figure 3. In the last part, the interference study was performed using blood plasma with L-lactic acid, uric acid, ascorbic acid, and glucose. All the data analysis was performed in Origin 8.5.0, including the normalization and statistical analysis.

## 3. Results and Discussion

### 3.1. Electrical Characterization of the Common-Gate Graphene-Based Field-Effect Transistors 

**Figure 4:**
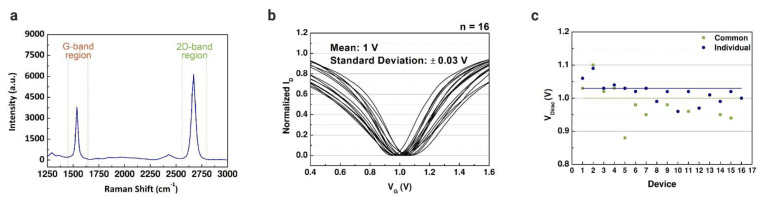
Characterization of common-gate graphene-based field-effect transistors. (**a**) Raman spectra (532 nm wavelength; G peak: 1537 cm^−1^ and 2D peak: 2670 cm^−1^) of the chemical vapor deposition (CVD)-grown graphene layers that was transferred using polydimethylsiloxane (PDMS) as supportive layer. (**b**) Normalized average transfer curves (n = 16) using 1× phosphate-buffered saline (PBS) (pH 7.4) as a buffer solution. (**c**) Comparison between the Dirac point voltage values of the common-gate GFET (mean = 1 ± 0.03 V) and the individual gate GFET (mean = 1.03 ± 0.06 V).

The measured transfer characteristics of the graphene-based devices were used to define reference points before the immobilization matrix formation and the lactate assays. In our previous work, a solution-gated GFET with a coplanar electrode array was developed for cancer diagnostics [50]. The intensity ratio of Raman spectra was measured (Figure 4a) to verify the quality of the graphene, where G and 2D peaks represent the number of graphene sheets. The properties of the graphene used in this study were already evaluated in our other works [34,38,50]. The graphene-based device introduced in this work is based on that coplanar structure. However, we modified the FET to have a common gate to cover all the microchannels connected to the electrode array. Before the lactate assays, all the channels were characterized using PBS solution in the channels; the drain current was measured while applying constant bias in the drain terminal and varying the gate bias. The properties of the two structures were compared through the results obtained by the transfer characteristics. Figure 4b illustrates the transfer curves measured for 16 common-gate GFET devices with a mean V_Dirac_ of 1 V (standard deviation (SD) of 0.03 V). The same was reproduced for the individual gate structure, with a mean V_Dirac_ of 1.03 V (SD of 0.06 V). The Dirac point voltage values of each structure are presented in Figure 4c. The common-gate structure presented a smaller standard deviation compared with the individual gate structure. However, there was no significant difference between the transfer curves of the structures, indicating that the simpler structure of the graphene-based device is enough to perform the assays to detect lactate. 

### 3.2. Detection of Lactate in Buffer Solution

After electrical characterization, all the microchannels were cleaned with deionized water and the lactate detection assay was performed. First, the enzyme was injected over the graphene surface into the eight microchannels. The device was placed in the probe station, and different concentrations of L-lactic acid (0.25 to 10 mM) were mixed with the LDH enzyme, considering that the reference line for bare graphene was measured with the buffer solution only. The concentration range was defined based on the reference values used to detect sepsis shock in blood samples, since lactate is a prognosis marker among septic shock patients [6,7]. 

**Figure 5:**
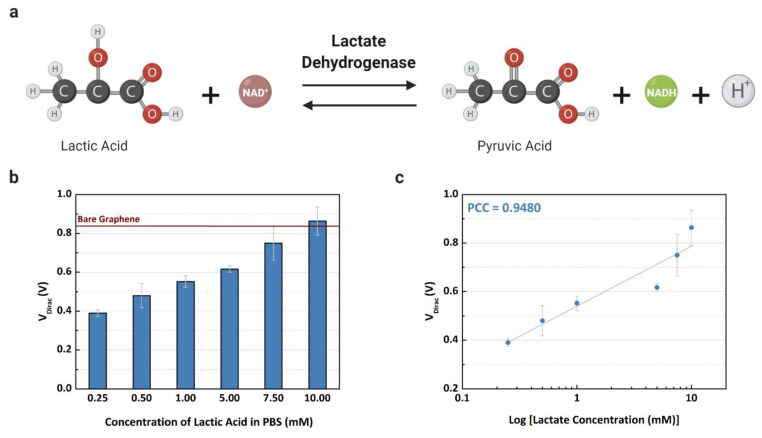
Experiments using the common-gate GFET device to detect L-lactic acid on buffer solution: (**a**) electrochemical reaction for the lactate assay; (**b**) the mean Dirac point (V_Dirac_) (n = 3) concentration of L-lactic acid (0.25 to 10 mM); and (**c**) the linear fit of the measured concentrations with a Pearson correlation coefficient of 0.9480 (R^2^ = 0.8735).

The Dirac point voltages were measured after the LDH enzyme catalyzed L-lactic acid, resulting in a product that doped the graphene surface with negative charges, as shown in Figure 5a. The values of the V_Dirac_ are shown in Figure 5b, demonstrating that the Dirac point voltage decreases following the increase of lactate concentration in the buffer solution. The measured V_Dirac_ values demonstrate a linear relationship with lactate concentration in Figure 5c, where the coefficient of determination is 0.9402 (R^2^ = 0.8549). Adding high concentrations of L-lactic acid in the channel with the presence of the LDH enzyme causes the production of more positive and neutral charges (NADH and H^+^), then the neutrality point is higher when the concentration increases [9]. Based on this relationship, the GFET device can detect the lactate level based on the charge concentration that is caused by the catalysis of the analyte in contact with the LDH enzyme. If the LOx enzyme is used instead of the LDH enzyme, the graphene-based sensor may be not able to detect lactate with the same sensitivity, considering the production of the hydrogen peroxide that has no partial charges. For this reason, lactate dehydrogenase was selected as an enzyme for this study.

### 3.3. Surface Modification and Stabilization

**Figure 6:**
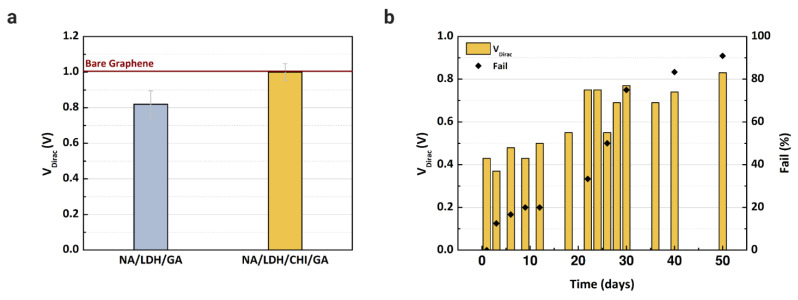
Dirac point voltage values of GFET devices: (**a**) Using Nafion (NA), chitosan (CHI), and glutaraldehyde (GA) to immobilize and stabilize the LDH enzyme over the graphene layer; (**b**) Dirac point voltage values and the failure percentage measured during the stabilization experiment to evaluate the device for up to 50 days after immobilization of the enzyme. A concentration of 5 mM of L-lactic acid in buffer solution was injected into each channel.

New devices were fabricated to evaluate the proposed immobilization matrix. It is necessary to reduce the number of layers over the active layer to avoid clogging the microchannels. Since Nafion and glutaraldehyde are essential to blocking the determined substances, we verified the performance of the enzyme with and without chitosan, since this material is an electron mediator that can improve the LDH enzyme immobilization capacity [8,19,48]. After the fabrication of the immobilization matrix, buffer solution was injected into two microchannels, one containing Nafion/lactate dehydrogenase/glutaraldehyde, and another with Nafion/lactate dehydrogenase/chitosan/glutaraldehyde. The transfer characteristics were measured using PBS solution for each matrix and compared with the reference V_Dirac_ obtained with bare graphene. Based on the results presented in Figure 6a, chitosan is essential to maintain the stability of the LDH enzyme, since the V_Dirac_ only shifted without the presence of chitosan. However, the matrix was still too thick for the microchannel; some devices experienced issues regarding the flow of analyte in the channel, even after drying them for more than 60 min. To overcome this clogging issue, a cleaning procedure with deionized water was repeated more than three times after the deposition of each component in the matrix. 

Considering the final immobilization matrix composed of Nafion, chitosan, LDH, and glutaraldehyde, stabilization of the enzyme was verified for up to 50 days while the devices were kept in a dry box at room temperature. In this study, we tested the storage of the devices in a refrigerator and a dry box, although the devices maintained in a refrigerator had no electrical response even after 2 days of storage. We decided to proceed only with the devices stored in the dry box. For each day measured, 5 mM of L-lactic acid were injected into the microchannel, and the signal response was recorded. The V_Dirac_ values of devices measured during the 50 days are shown in Figure 6b, where the graph includes the failure percentage of the devices. The V_Dirac_ increased greatly after 12 days of measurement, as did the number of failed devices. The instability of the enzyme can be considered a drawback of the method presented in this study, and it is necessary to evaluate a new storage approach to guarantee the enzyme function for at least 6 months, since the device will be adapted as a point-of-care sensor in future study.

### 3.4. Detection of Lactate in Human Plasma 

**Figure 7:**
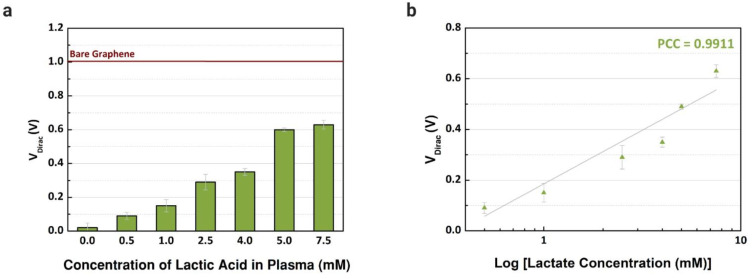
Detection of different concentrations of L-lactic acid in human samples. (**a**) Dirac point voltage values (n = 3) for blood plasma samples treated with L-lactic acid (0 to 7.5 mM). (**b**) Comparison between the shift of Dirac point voltage by concentration of lactate (Pearson coefficient correlations: 0.9911).

Previously, the performance of common-gate GFET devices was verified using the proposed immobilization in buffer solutions with different concentrations of L-lactic acid. Then, blood plasma was used as an electrolyte to confirm the capability of the device to detect lactate in real human samples. First, blood plasma was injected (10 µL) into the channels after being mixed with different concentrations of lactate, from 0.25 to 7.5 mM, based on the reference values in the literature considering clinical emergencies where the level of lactate is abnormal [28]. As observed in Figure 7a, the V_Dirac_ increased from the lowest to the highest concentration in blood plasma samples. For each concentration, three channels were used to obtain the error bars. The variation of the V_Dirac_ for the selected biological fluid is presented in Figure 7b, and linear responses are observed, with a Pearson correlation coefficient of 0.9911 (R^2^ = 0.9787, n = 3). Compared with the works in the literature that also used human plasma samples, the linear response observed with the graphene-based device was similar or superior, and we also investigated a wider range of concentrations [28,51]. Besides plasma, we focused on serum samples, however, plasma presented a higher sensitivity while detecting lactate, since the sensitivity error was smaller when comparing the slope of the curves with our previous experiments using samples of human serum.

### 3.5. Interference Study

New experiments were performed to evaluate the interference from electroactive species in the human samples [20,21]. The electroactive interferents, uric acid or ascorbic acid, contained in blood were selected considering that the oxidation of these substances can happen in the presence of the enzyme [9,21,22]. In addition, glucose was included because it can cause a doping effect over the active layer. First, V_Dirac_ of the buffer solution was used as a reference point. The plasma sample containing 7.5 mM of L-lactic acid was injected into the same microchannel as when the transfer characteristics were measured; this procedure was repeated for each interference species with concentrations that were considered for a normal blood sample from a healthy human. 

**Figure 8:**
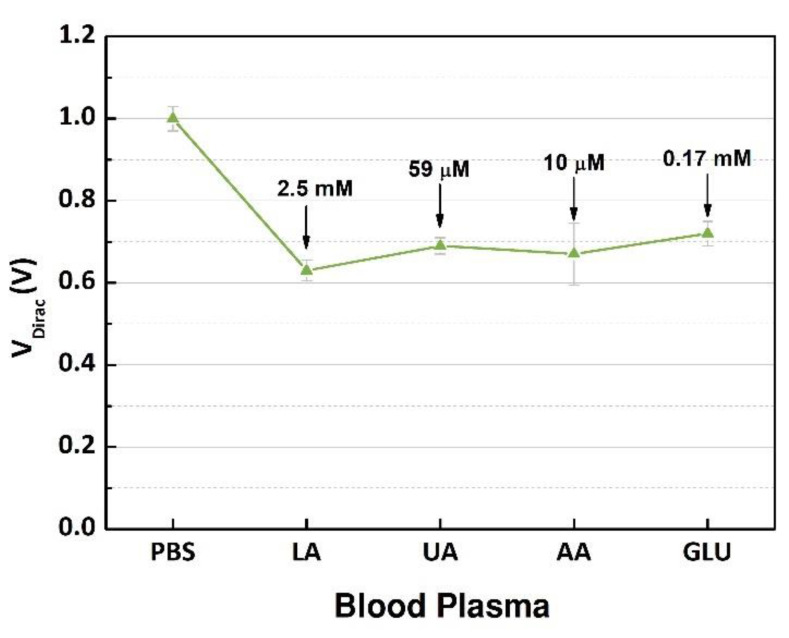
Measurement of the Dirac point voltages (n = 3) of PBS, plasma sample containing 7.5 mM of L-lactic acid (LA), and after injection of each interference biochemical (uric acid (UA), ascorbic acid (AA), and glucose (GLU)).

The GFET devices were measured, and the output signals were analyzed, and the V_Dirac_ values are presented in Figure 8. The highest variation (0.37 V) occurred after the blood plasma sample with 2.5 mM of lactate was inserted into the microchannel, indicating that the products of the enzymatic reaction doped the graphene structure. However, after the interference substances were mixed into the sample one by one, there was no relevant variation in the neutrality points of graphene; the maximum shift around 0.09 V was caused by glucose. The interference study evaluated the selectivity of the device regarding the target, lactate, that was successfully detected, and the other substances had no significant effect on the charge concentrations over the graphene. These results demonstrated the capability of the components contained in the immobilization matrix to block the interferents and select the target analyte. Additionally, compared to other devices using the same enzyme [8,19,20], our device had a higher performance regarding the selectivity while dealing with high concentrations of interferents, which is ideal while detecting the NADH contained in the product of the catalytic reaction due to the high number of electroactive species. Additionally, the concentration range was wide to guarantee the detection of high levels of lactate in clinical conditions where a rapid and accurate diagnosis is necessary. 

## 4. Conclusions

We demonstrated in this study a graphene-based biosensor to provide rapid quantitative measurement of lactate in the buffer solution and blood plasma. Determination of L-lactic acid was performed by measuring the transfer characteristics of graphene in a field-effect transistor structure with different concentrations of the analyte. Different materials were tested for the immobilization matrix to guarantee the stabilization and selectivity of the device; the best combination includes Nafion, chitosan, lactate dehydrogenase, and glutaraldehyde. The proposed immobilization matrix was structured considering the role of each biochemical to improve the performance of the graphene-based sensor. After the measurements, a linear response was observed for all the samples, including the blood plasma, with different concentrations (from 0 mM to 7.5 mM). The capability of detection of lactate at high concentrations (up to 7.5 mM) indicates that the proposed device can be implemented for POC applications for different disorders, such as septic shock and left ventricular failure. The presence of chitosan was essential to maintain the stability of the LDH enzyme, which was evaluated through the electrical measurements after storing the GFET devices at room temperature in a dry box for up to 50 days. After 12 days, there was a significant shift of V_Dirac_, indicating a limitation of this enzyme with the respective immobilization matrix. Additionally, different interference substances (uric acid, ascorbic acid, and glucose) were injected into the GFET devices during the lactate assays to verify the selectivity of the sensor. The variation of V_Dirac_ was significant after injection of the plasma sample with 7.5 mM of lactate considering the reference point (PBS solution), and the maximum shift observed was 0.09 V among interference substances. Considering the results obtained in this study, graphene demonstrated the potential to detect L-lactic acid in human fluids without the interference of other biochemicals. To the best of our knowledge, a GFET device to detect the concentrations of lactate in human samples had not been reported, also considering the combination of elements in the immobilization matrix. With the implementation of low-cost materials to our proposed method, it is possible to detect lactate in a fast and precise diagnostic method for clinical applications taking advantage of the properties of the graphene-related materials, e.g., graphene oxide and reduced graphene oxide. The next step of this study is to include two new analytes to increase the prediction of a sepsis shock by developing a flexible, low-cost, and disposable chip for a point-of-care device with a rapid and accurate response.

## Data Availability

Not applicable.
